# The Deadly Contaminant: A Case of Staphylococcus lugdunensis Endocarditis

**DOI:** 10.7759/cureus.49748

**Published:** 2023-11-30

**Authors:** Sammudeen Ibrahim, Saint-Martin Allihien, Inemesit Akpan, Olawole Akinboboye, Kofi D Seffah

**Affiliations:** 1 Graduate Medical Education, Piedmont Athens Regional Medical Center, Athens, USA; 2 Internal Medicine, Piedmont Athens Regional Medical Center, Athens, USA; 3 Internal Medicine, Phoebe Putney Memorial Hospital, Albany, USA

**Keywords:** highly virulent, coagulase-negative staphylococcus, contaminant, staph lugdunensis, infective endocarditis

## Abstract

The incidence of infective endocarditis (IE) has been on the rise since it was first reported a century ago, and the associated mortality remains unchanged despite advances in medical and surgical management. To diagnose IE, the modified Duke criteria are used, which rely on isolating the causative organism. However, this can be challenging if the micro-organism is considered a contaminant. Staphylococcus lugdunensis (SL) is one such organism. In this case, an elderly female presented with intermittent chest pain, palpitation, and diaphoresis, for which she underwent left heart catheterization. Her hospital course was complicated by persistent fever and night sweats, leading to blood cultures isolating methicillin sensitivity. It was initially reported as a contaminant. However, an extensive workup was unremarkable, and a transthoracic echocardiogram was done, which revealed tricuspid vegetations with moderate regurgitation. The patient was treated with cefazolin, repeat cardiac imaging at the end of treatment revealed no vegetation, and the patient remained asymptomatic. Despite being associated with fulminant IE with higher mortality than Staphylococcus aureus (S. aureus), which requires surgical management in most cases, SL is still often reported as a contaminant. Isolation of SL should warrant further investigation beyond mere contaminants, and prompt treatment should be initiated in the appropriate clinical scenario to avoid poor outcomes.

## Introduction

Since infective endocarditis (IE) was first reported by Sir William Osler over a century ago, it has been described as primarily affecting the endothelial surface of the heart valves with various clinical consequences on multiple organ systems [1]. Despite our understanding of the disease, the incidence of IE has continued to soar. While the annual incidence was previously estimated at 5 to 7 cases per 100,000 person-years between 1970 and 2000, it increased to about 15 cases per 100,000 population in 2011 [2,3]. In addition, even with the advancement in medical and surgical management, the mortality rate has remained unchanged at around 25%, which has been attributed to diagnostic and treatment delays [4].

IE is diagnosed with the modified Duke criteria, which has a sensitivity approaching 80%. Aspects of these criteria depend on the isolation of a typical causative organism. However, this is sometimes challenging if the organism is not typically associated with the disease or is often considered a contaminant [5]. One such organism is Staphylococcus lugdunensis (SL). Herein, we present a case of SL bacteremia complicated by IE. Initially thought to be a contaminant, it was further evaluated due to persistent symptoms, and the appropriate treatment was initiated. This case highlights the need to thoroughly evaluate cases of SL bacteremia beyond mere contaminants, where necessary, to avoid adverse clinical outcomes associated with therapeutic delays.

## Case presentation

**Figure 1:**
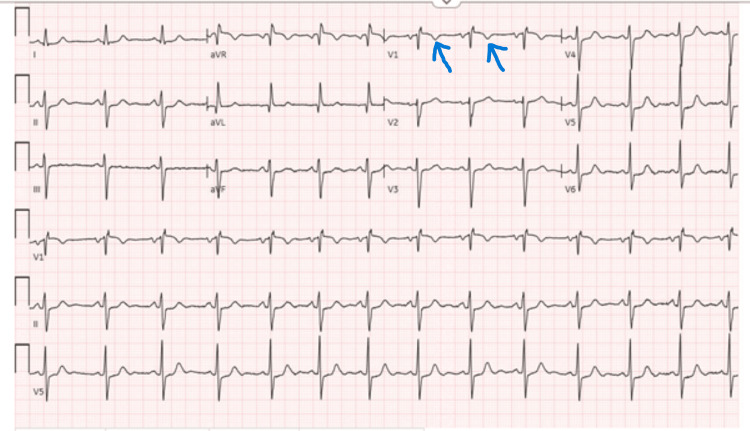
Electrocardiogram showing normal sinus rhythm with non-specific ST changes (blue arrows)

A 68-year-old female with a medical history of hypertension and anxiety presented to the emergency department (ED) on 3/21/2023 with a complaint of intermittent chest pain. The pain radiated to her left arm and was associated with diaphoresis and palpitations. She denied any other symptoms. Upon examination, the vitals were stable, with a temperature of 98.2 ^0^F (36.8 ^0^C). The chest wall was non-tender, and the first and second heart sounds were present without murmurs. The initial laboratory investigations showed a white blood cell count of 6.0 x 10^3^ µL, hemoglobin of 13.2 g/dL, and platelet count of 234 x 10^3^ µL. Urinalysis, thyroid-stimulating hormone test, and a complete metabolic panel were unremarkable. Highly sensitive troponins at 0, 2, and 6 hours were 12, 224, and 384 ng/mL, respectively. The electrocardiogram showed a sinus rhythm with nonspecific ST changes (Figure 1). Given the persistent chest pain and rising troponins, there was an initial concern for non-ST-elevation myocardial infarction (NSTEMI), and the patient was given 400 µg of nitroglycerin and aspirin 324 mg, and an unfractionated heparin drip was started. Cardiology was consulted due to persistent chest pain, and the patient underwent left heart catheterization (LHC) via femoral access, which revealed non-obstructive coronary artery disease.

**Figure 2:**
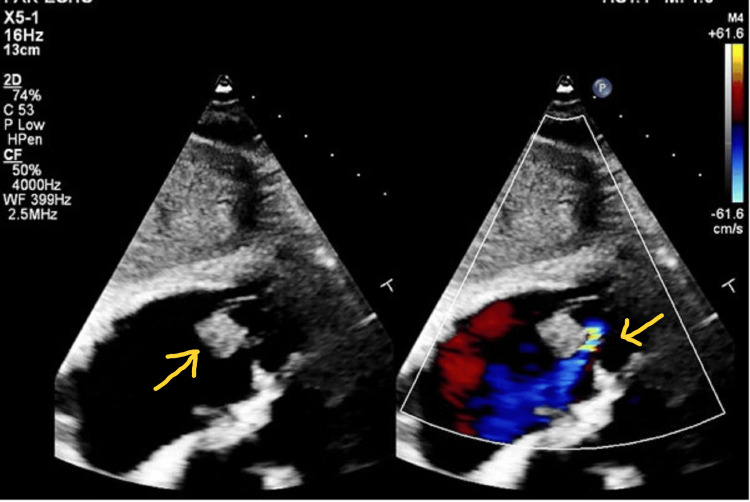
Transthoracic echocardiogram showing multiple tricuspid valve vegetations and tricuspid regurgitation (yellow arrows)

On the second day post-LHC, the patient experienced multiple febrile episodes ranging between 100.8 ^0^F and 101.3 ^0^F. The patient was re-examined for any obvious source on the skin, ear, nose, and throat, which were unremarkable. A complete blood count (CBC), respiratory panel, including nucleic acid amplification of common viral and bacterial pathogens, rapid strep test, throat swab culture, urinalysis with reflex culture, and blood cultures were taken. The patient also had a repeat chest X-ray and lower extremity Dopplers and was started on empiric antibiotics with intravenous vancomycin 25 mg/Kg twice daily and intravenous cefepime 1 g twice daily. All investigations remained unremarkable except the blood culture, which returned positive for SL in one out of two sets but was reported as a contaminant. A repeat blood culture was taken but returned negative, likely a false-negative from antibiotics exposure. The patient continued to experience episodes of fever and night sweats through day 4 post-LHC. Infectious disease (ID) was consulted and they recommended Fungitell® (Associates of Cape Cod, Inc., East Falmouth, MA, USA) and a transthoracic echocardiogram (TTE) to evaluate for fever of unknown origin, especially given the patient had a recent LHC with a reported gram-positive bloodstream contaminant. The TTE showed moderate tricuspid regurgitation and new multiple tricuspid valve vegetations with the largest measuring 1.1 x 1.3 cm without perivalvular abscess (Figure 2). The Fungitell, however, returned negative. An impression of possible IE was made per the modified Duke criteria and the antibiotics were continued with ID requesting for a susceptibility report to be run on the blood culture sample that isolated SL.

The susceptibility report revealed methicillin sensitivity with a negative Mec A/C gene on polymerase chain reaction. The antibiotics were de-escalated to IV cefazolin 2 g every 8 hours, and she was discharged to continue for a total of six weeks via a peripherally inserted central catheter (PICC) line with an excellent clinical response. Interval cardiac imaging at the end of treatment revealed no tricuspid vegetation, and the patient reported no further symptoms.

## Discussion

SL is a gram-positive coagulase-negative staphylococcus (CoNS), which is typically a skin flora on the lower parts of the body, particularly the perineal area [6]. As a CoNS, SL is often dismissed as a contaminant (defined as the isolation of a CoNS in one out of two blood culture sets) when isolated from clinical specimens as seen in our patient scenario [7,8]. This often leads to a delay in diagnosis and treatment resulting in poor clinical outcomes [7]. The clinical significance of blood culture-positive SL has therefore been a topic of discussion over the last 2 decades [9]. Although the incidence of SL bacteremia has been estimated at 5.6 patients per 100,000 hospital admissions, only 1.3 patients per 100,000 admissions represent clinically relevant bacteremia [9]. A retrospective cohort study and systematic review also reported the incidence of IE at 6.3% to 27% in patients who had one or more positive blood cultures for SL [10].

Unlike other CoNS, however, SL shares many virulent characteristics with Staphylococcus aureus (S. aureus) [11]. SL encodes virulence factors for adhesion, cytotoxicity, and innate immune evasion. This includes fibrinogen-binding protein (fbl), a homolog of Staphylococcus aureus clumping factor A, and von Willebrand factor, which facilitates bacterial adherence and endovascular invasion by an uncharacterized mechanism [11,12]. Therefore, like S. aureus, SL typically causes infections with an aggressive clinical course, including life-threatening endocarditis, myocarditis, and central nervous system infections, leading to cerebrovascular accidents [13,14]. More recent studies have also reported cases of fatal septic emboli associated with SL bacteremia requiring mechanical thrombectomy with or without a co-existing IE [15,16].

Since its isolation in 1988, SL endocarditis has been described as an aggressive disease characterized by rapidly growing large vegetations, abscess formation, valvular destruction, and high mortality requiring surgical intervention [17,18]. Multiple other literatures have reported SL causing aggressive diseases with fatal clinical outcomes despite antibiotic therapy; thus, emphasizing the high virulence of this organism [9,19-21]. SL has also been described as causing hospital-acquired infections [19]. In one study, most patients with SL infections either had an existing catheter or underwent an invasive procedure [19]. They also had comorbidities that predisposed them to frequent hospitalizations [19]. Our patient did not have multiple comorbidities that exposed her to frequent hospitalizations, but she underwent an LHC, which most likely was the risk factor for SL bacteremia with resultant IE.

Despite the significant virulence associated with SL, unlike the other CoNS, it is susceptible to a wide range of antibiotics and resistance to methicillin antibiotics has been far less common due to the absence of the mecA gene [22,23]. Owing to this and based on the susceptibility report, our patient was eventually treated and discharged on intravenous cefazolin for six weeks, leading to a favorable clinical outcome. Nonetheless, a prospective cohort study reported that more than 50% of cases of SL endocarditis required surgical intervention representing a higher surgical rate than S. aureus endocarditis [24]. This was thought to be the result of delayed diagnosis and treatment [24]. Due to the associated poor outcome, early surgical intervention and aggressive intravenous antibiotic therapy have been shown to significantly reduce mortality in patients with a complicated disease [25].

In our patient, the early diagnosis with a high index of suspicion was likely crucial in altering the naturally aggressive course of the organism. Therefore, when SL is isolated in blood cultures, it is important to investigate further in the appropriate clinical setting and treat accordingly to prevent any possible complications and mortalities associated with delayed therapy.

## Conclusions

SL is an increasingly common cause of rapidly progressive endocarditis, which is sometimes reported as a contaminant in blood samples. Early diagnosis and treatment are essential to mitigate the adverse clinical outcome from SL endocarditis. However, how often SL causes clinically significant disease when isolated in blood culture samples remains an area of continued discussion. Therefore, larger observational studies are necessary to assess the frequency of invasive disease, including IE, in patients with blood culture-positive SL and to understand the factors associated with the development of a clinically relevant disease.
